# VIRUS-MVP: a framework for comprehensive surveillance of viral mutations and their functional impacts

**DOI:** 10.1093/nargab/lqaf132

**Published:** 2025-10-07

**Authors:** Muhammad Zohaib Anwar, Ivan S Gill, Madeline Iseminger, Anoosha Sehar, Emma J Griffiths, Damion Dooley, Jun Duan, Khushi Vora, Gary Van Domselaar, Fiona S L Brinkman, Paul M K Gordon, William W L Hsiao

**Affiliations:** Centre for Infectious Disease Genomics and One Health, Faculty of Health Sciences, Simon Fraser University, Burnaby, V5A 1S6 BC, Canada; Centre for Infectious Disease Genomics and One Health, Faculty of Health Sciences, Simon Fraser University, Burnaby, V5A 1S6 BC, Canada; Centre for Infectious Disease Genomics and One Health, Faculty of Health Sciences, Simon Fraser University, Burnaby, V5A 1S6 BC, Canada; University of British Columbia, Vancouver, V6T 1Z7 BC, Canada; Centre for Infectious Disease Genomics and One Health, Faculty of Health Sciences, Simon Fraser University, Burnaby, V5A 1S6 BC, Canada; Centre for Infectious Disease Genomics and One Health, Faculty of Health Sciences, Simon Fraser University, Burnaby, V5A 1S6 BC, Canada; Centre for Infectious Disease Genomics and One Health, Faculty of Health Sciences, Simon Fraser University, Burnaby, V5A 1S6 BC, Canada; Centre for Infectious Disease Genomics and One Health, Faculty of Health Sciences, Simon Fraser University, Burnaby, V5A 1S6 BC, Canada; Centre for Health Genomics and Informatics, Cumming School of Medicine, University of Calgary, Calgary, T2N 1N4 AB, Canada; National Microbiology Laboratory, Public Health Agency of Canada, Winnipeg, R3E 3R2 MB, Canada; Department of Molecular Biology and Biochemistry, Simon Fraser University, V5A 1S6 Burnaby, Canada; Centre for Health Genomics and Informatics, Cumming School of Medicine, University of Calgary, Calgary, T2N 1N4 AB, Canada; Centre for Infectious Disease Genomics and One Health, Faculty of Health Sciences, Simon Fraser University, Burnaby, V5A 1S6 BC, Canada; Department of Molecular Biology and Biochemistry, Simon Fraser University, V5A 1S6 Burnaby, Canada

## Abstract

As viruses evolve, they accumulate genetic mutations that can influence disease severity, transmissibility, and the effectiveness of vaccines and therapeutics. Real-time tracking of viral mutations and their functional impacts is essential to understand these changes and assess their implications for public health responses. VIRUS-MVP is an interactive, portable platform designed for the comprehensive surveillance of viral mutations. Initially developed for SARS-CoV-2, it now fully supports mpox and is expanding to include influenza and RSV. The platform links viral mutations to functional annotations, providing insights into their predicted effects on viral infectivity, immune evasion, and protein functionality. It features an interactive interface for visualizing mutation distributions, a modular and reproducible genomics workflow, and a curated annotation resource that captures known impacts on viral proteins and host interactions. Users can also import custom functional annotations to tailor analyses to specific research needs or emerging pathogens. Developed collaboratively with public health and academic partners, VIRUS-MVP enhances understanding of viral evolution and its public health impact by bridging genomic data with biological insights. The platform is open-source, adaptable, and accessible on GitHub.

## Introduction

During the COVID-19 pandemic, genomic epidemiology offered unprecedented insights into the evolution and transmission dynamics of SARS-CoV-2, the virus responsible for the pandemic. In previous viral outbreaks, genomic epidemiology was mainly applied retrospectively, i.e. employing genomic data for analyses conducted after the events, or in real-time for a specific region [1–3]. However, the advent of COVID-19 marked a paradigm shift, enabling real-time surveillance due to the unprecedented scale of genomic data generation, sharing, and analysis [4, 5]. This transition underscores the transformative potential of genomic epidemiology in responding to pandemics. As the world navigates the post-pandemic landscape, the importance of continued surveillance for circulating viruses like influenza, mpox, and respiratory syncytial virus (RSV), alongside monitoring for emerging pathogens, cannot be overstated [6, 7]. The global increase in public health genomics capacity achieved during the COVID-19 pandemic positions us to confront future infectious disease threats more effectively. As part of that capacity building, we developed COVID-MVP [8], a platform designed to monitor SARS-CoV-2 population structures, mutations, and real-time functional impacts of mutations. COVID-MVP bridged a significant gap in genomic surveillance and public health response by connecting the mutations harbored by variants to their possible functional impacts [8].

RNA viruses exhibit moderately high mutation rates due to the error-prone nature of their replication machinery [9]. The accumulation of mutations leads to a diverse viral population, allowing the viruses to adapt to changing environments and potentially evade host immune responses. During the COVID-19 pandemic, several visualization dashboards and analytical workflows were developed to track the prevalence of mutations: popular examples include CovMT [10], CoV-Spectrum [11], CovRadar [12], and Outbreak.info [13, 10–13]. Such tools provide valuable insights into the mutational landscape of SARS-CoV-2, summarizing mutations by geographical location, sampling date, and disease severity. They also offer data on variant proportions, common mutations, and associated hospitalization and mortality probabilities. COVID-MVP distinguished itself from other mutation-tracking tools by connecting mutations to their functional impacts in real time. This capability was crucial for assessing the evolving viral population and epidemiological characteristics, such as transmissibility, disease severity, immune escape, and the effectiveness of vaccines and therapeutics [14].

The need for ongoing genomic surveillance becomes increasingly paramount as we transition from pandemic response to preparedness. Building upon the development of COVID-MVP, we here present VIRUS-MVP, a virus-agnostic modular and extensible toolkit comprising three standalone components, each contributing to the platform’s flexibility, portability, and adaptability. The visualization component is a user-friendly interactive webpage powered by Python (using Plotly and Dash) and JavaScript. The second component is the genomics workflow, implemented in the Nextflow workflow management framework [15] and following the nf-core guidelines [16]. The workflow processes genomic data in a modular, reproducible, and scalable manner, offering a plug-and-play approach for customization and selection of modules based on pathogen characteristics. Finally, the third component is the functional annotation database, named Pokay. Pokay is a manually curated database integrated into the genomic variant data to annotate the mutations and their functional impact.

Given the high mutation rates in many viruses and the need to identify rare alleles, a tool like VIRUS-MVP is essential for ongoing genomic surveillance and public health response. VIRUS-MVP can be tailored for different viruses, catering to each pathogen’s specific characteristics. This design enables real-time monitoring of population structures, mutations, and their functional impacts. By harnessing the power of genomic epidemiology, VIRUS-MVP represents a significant advancement in our ability to understand viral sequencing data and helps public health practitioners, epidemiologists, and academic researchers by quickly summarizing the functional significance of viral mutations. All three components of the framework are available open-source (see the “Availability and deployment” section) under the Massachusetts Institute of Technology (MIT) and GPL-3.0 licenses.

## Materials and methods

### Genomics data and formats

The VIRUS-MVP genomics workflow accepts assembled contigs (e.g. from clinical specimens) or paired-end reads (e.g. from wastewater sampling) along with an optional metadata file (in CSV format) and outputs a collection of identified mutations as a Genome Variant Format (GVF) file. The metadata file can include multiple variables such as sampling date, geographic location, and/or lineage information. These variables can be used as criteria for grouping samples. Currently, the genomics workflow supports processing genomic data from two viruses: SARS-CoV-2 and mpox.

The workflow supports the ability to choose from various compatible tools for a given step; for instance, more than one variant calling tool is available to choose from based on the sequence data type and sequencing depth [17]. This flexibility, however, adds interoperability challenges as different tools have different output formats. To address this challenge, we use standardized data formats and have developed helper functions to convert the varying analytic tool output formats into these standard formats. We have used VCF (Variant Call Format) and GVF (Genome Variation Format) files as the standard formats for variant data. VCF is a widely adopted community standard for representing genomic variants, thus promoting data interoperability and exchange. GVF provides a structured encoding of genomic variations along with associated functional annotations.

We are developing VIRUS-MVP to support additional specific pathogens such as RSV and influenza. Additionally, VIRUS-MVP’s modular architecture and developer documentation allow end users/developers with modest coding expertise to adapt the workflow to other viruses rapidly.

### Annotation sources

VIRUS-MVP can utilize multiple data sources to annotate viral mutations. Primary data sources include the following:

NCBI: The genomics workflow utilizes NCBI GenBank [18] to annotate each virus’s genomic features [including genes and coding sequences (CDS), noncoding regions, and protein products], and adding context to the mutations they harbor.

Problematic sites identification: Sequencing errors, contamination, and hypermutable sites must be identified to ensure the accuracy of downstream analyses, such as phylogenetic reconstruction, variant interpretation, and genomic epidemiology. We have incorporated information regarding problematic sites identified in SARS-CoV-2 genomes using data from De Maio *et al.* [19]. These observations are marked as either “Mask” or “Caution” based on the severity of their impact on the analysis. Each site is further labeled with a more specific potential problem. A custom Python script is integrated into the workflow to parse the observations and integrate them with the mutations observed in the sequencing data analyzed.

Pokay functional database: At the heart of this framework is the continuous curation of functional annotations and their connection to mutations. This process is essential for understanding how genetic changes impact the behavior of viruses, aiding in our comprehension of their evolution and disease-causing mechanisms. For the COVID-19 pandemic response, one of our primary goals was to curate functional annotations for mutations in the SARS-CoV-2 genome. Since then, Pokay has also been extended to mpox and influenza annotations, broadening its scope to encompass a wider range of viral pathogens.

For COVID-19 surveillance, Pokay has served as one of the most comprehensive resources for understanding the functional implications of SARS-CoV-2 mutations. Pokay is hierarchically structured, with functional categories paired with specific features of the viral genome. For instance, categories such as “S_convalescent plasma escape” describe functions related to convalescent plasma escape specifically associated with mutations in the SARS-CoV-2 S gene (encoding the spike protein). Similarly, categories like “RdRp_pharmaceutical_effectiveness.txt” capture mutations in the RdRp mature peptide associated with pharmaceutical effectiveness. Within each functional category, specific functions and associated mutations are curated. Additionally, comprehensive information, including the source(s), citation(s), and URL(s) of relevant studies, is listed to facilitate further exploration and verification. Pokay’s hierarchical design disentangles the complex relationships between mutations and their functional consequences. Notably, a mutation may be implicated in single or multiple functions, and conversely, a single functional impact may be associated with individual mutations or combinations thereof. A Python script has been provided with the repository to merge functional data from all categories within Pokay, generating a structured TSV file, which is then used for integrating functional information into the GVF files, facilitating visualization and surveillance reporting. Pokay comprises over 40 unique functional categories and contains >4000 functional annotations for over 1000 mutations, individually or in combination. Pokay is updated regularly with the latest information on mutations and their associated functions. This undertaking involves a comprehensive literature search and curation to ensure that Pokay remains a reliable and up-to-date resource for researchers and stakeholders.

Furthermore, we are working on ontologizing the functional annotations, mutations, and additional data using a controlled vocabulary. This ongoing effort is the subject of a separate study, which will also include our collaboration with the Coronavirus Infectious Disease Ontology (CIDO) to integrate all mutations into CIDO [20, 21]. This initiative aims to enhance interoperability, data integration, and semantic consistency across various resources.

### Data standards

We adhere to a range of established genomic data standards to ensure consistency, interoperability, and accuracy in data representation, analysis, and sharing, including, as a notable example, the use of Human Genome Variation Society (HGVS) nomenclature [22]. The HGVS format provides a standardized and internationally recognized method for describing mutations, facilitating precise annotation and interpretation of mutations. The adoption of HGVS ensures that genomic variants are uniformly and consistently represented across datasets and analyses, facilitating cross-sector integration and interpretation [23].

A key guiding principle in VIRUS-MVP’s development is adherence to the FAIR (Findable, Accessible, Interoperable, and Reusable) data principles [24]. By making genomic data and its associated metadata findable through standardized ontologies, accessible through open data portals, interoperable via structured formats like JSON and YAML, and reusable by ensuring transparent data provenance, VIRUS-MVP enables robust data sharing and reusability. These principles not only support scientific reproducibility but also facilitate collaboration across diverse research communities and public health agencies. To further support these goals, a dedicated “Run Info” section has been added to all public instances of VIRUS-MVP, clearly outlining data provenance, processing parameters, reference genomes, dataset size, and download links for annotated mutation data in machine-readable format.

## Results

### Implementation

VIRUS-MVP consists of three main components: [1] a visualization app, [2] a genomics workflow, and [3] a functional annotation framework that are tightly integrated but also independently usable. The system is designed to support two primary modes of use:

Web-based interactive mode: All three components are deployed within a containerized framework that allows users to upload viral genome sequences (e.g. FASTA files) through the web interface. This triggers the genomics workflow and annotation pipeline in real time, making the results immediately available in the heatmap-based dashboard for exploration and comparison with the pre-calculated datasets. This mode is optimized for small- to moderate-sized datasets and enhances accessibility for users without local computational resources.

Standalone and batch processing mode: Genomics workflow is also available as a modular standalone workflow for command-line use. This enables advanced users to process large-scale datasets (tested for 600 000 + SARS-CoV-2 sequences), customize parameters, or integrate the system into existing bioinformatics pipelines.

This hybrid architecture ensures that VIRUS-MVP is accessible to a broad user base, ranging from public health scientists seeking quick insights via the web to power users performing large-scale viral genomic analysis in local or cloud computing environments. The system’s modular design also facilitates reuse and extension by the community.

Below, we describe the design of each component individually and how they function independently and together as a system.

#### Visualization design

The visualization component is designed to be customizable, as the visualization is rendered by the configuration file produced as part of the genomics workflow. Visualization is implemented almost entirely in Python (using the graphing library Plotly and the visualization framework Dash) and is supported by JavaScript components. Currently, two different implementations are available: one supports real-time processing of user-uploaded data (see the “Design of the genomics workflow” section below), enhancing its suitability for data analysis and dashboarding, while the other one, without the upload function, is more suitable for visualizing the pre-processed data for public-facing surveillance dashboards. Both versions are available for local and cloud deployment and can be cloned from the GitHub repository (see the “Availability and deployment” section).

##### Heatmap

**Figure 1.**
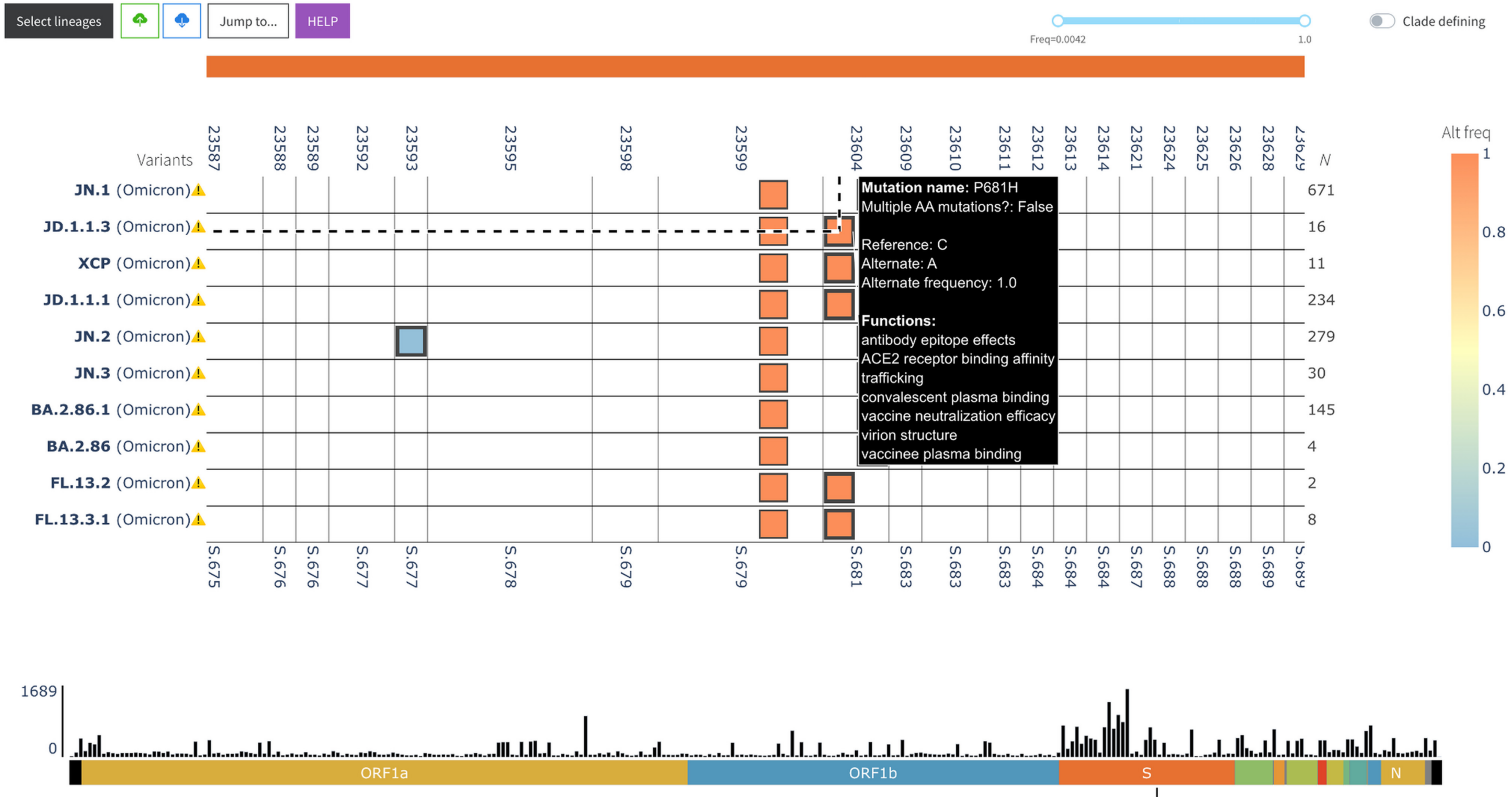
Screenshot of VIRUS-MVP web interface with SARS-CoV-2 sequences grouped into lineages. The central heatmap encodes mutation frequency with functional information available on hover. The bottom histogram shows the distribution of mutations across the genome.

The visualization component is an interactive web application centered on a heatmap that encodes the frequency of mutations at a given site across different groups (e.g. SARS-CoV-2 lineages, as shown in Fig. 1, or user-defined groupings). The heatmap has multiple axes, encoding the following attributes:

Top: nucleotide position of mutation with respect to the reference viral genome, e.g. NC_045512.2 for SARS-CoV-2 and NC_063383.1 for mpox (or user-provided reference genome).

Bottom: amino acid position of the mutation within its gene, or if the mutation is in an intergenic region, the number of amino acid positions upstream/downstream of the closest gene.

Left: sample ID (wastewater mode) or sample groups based on different criteria, e.g. time, region, or lineage. In the case of lineages, variant status is also added to the group, e.g. Variants of Concern.

Right: number of sequences or paired-end reads in each group.

**Figure 2.**
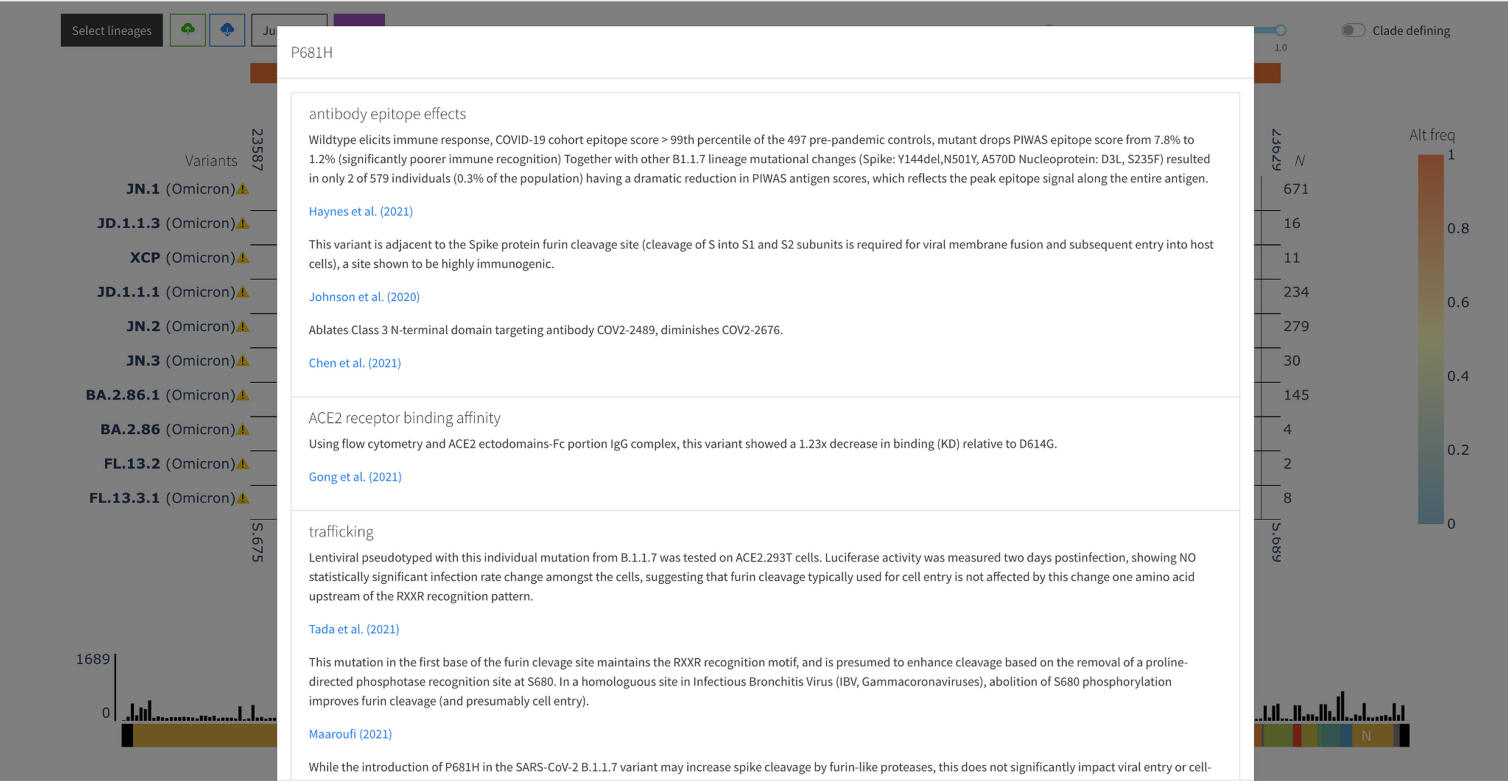
The modal opens when a user clicks on a heatmap cell with functional annotations in VIRUS-MVP. A description of each function and its primary source is provided as clickable links.

Heatmap cells encoding insertion and deletion mutations are annotated with unique markers, and cells representing mutations with known functions are represented with a thicker border. Users can hover over the heatmap cells for more information on each mutation. Whether the mutations have associated functions, users can also click on the cells to open a modal with a description and source of each function, as shown in Fig. 2.

##### Histogram

Under the heatmap, a histogram encodes the total number of mutations across all visualized groups per 100 nucleotides with respect to the reference. This secondary visualization summarizes the distribution of mutations across the genome, as shown in Fig. 1. The histogram also serves as a navigation aid: by clicking on a gene label below the histogram, users can fast-scroll the heatmap to the start of that gene, allowing efficient exploration of large genomes.

##### Editing the visualization

The visualization can be edited in several ways. A “select groups” button at the top-left of the application opens a modal that allows users to filter or change the order of groups/lineages visualized in the heatmap. A “clade-defining” switch enables users to quickly filter out mutations with <75% frequency. We define the clade-defining mutations similar to the Outbreak.info [13]. However, a frequency slider to the left of this switch provides greater granularity for filtering mutations by frequency if necessary.

##### Downloading and uploading data

An “upload” button to the right of the “select groups/lineages” button allows users to upload known variants as a VCF file. Alternatively, users can upload a collection of sequences as a FASTA file. The genomics workflow processes the input data and presents the result alongside the existing data as a heatmap (currently supported for SARS-CoV-2 only). A “download” button is also available that allows users to export a ZIP file containing the surveillance reports for each group (see *Surveillance Reports* below).

#### Functional annotation framework

In addition to the Pokay function database already described, we have developed a framework that allows the integration of annotations from additional sources. This process required the development of a standardized, generic template that allows external annotations to be integrated with variant calls, either in conjunction with or in place of Pokay annotations. The development of this template posed significant challenges, including the need for extensive data standardization, controlled vocabulary, and consistent formats, such as the HGVS nomenclature. Standardizing the format and vocabulary of external annotations was crucial to ensure interoperability and compatibility with existing genomic analysis pipelines. This process involved meticulous data curation and harmonization efforts to reconcile differences in annotation styles and terminology used across different sources.

To facilitate the integration of external annotations, we developed a Mutation Functional Annotation Contextual Data Specifications package (see the “Availability and deployment” section). Users can use this template to add functional information using a controlled vocabulary and pick lists and integrate them into their analyses. To validate user-developed annotations, we have developed a DataHarmonizer [25] template for VIRUS-MVP functional annotation. This harmonization process enhances data quality and creates interoperability between different systems, facilitating collaborative research efforts and cross-study comparisons.

#### Design of the genomics workflow

**Figure 3.**
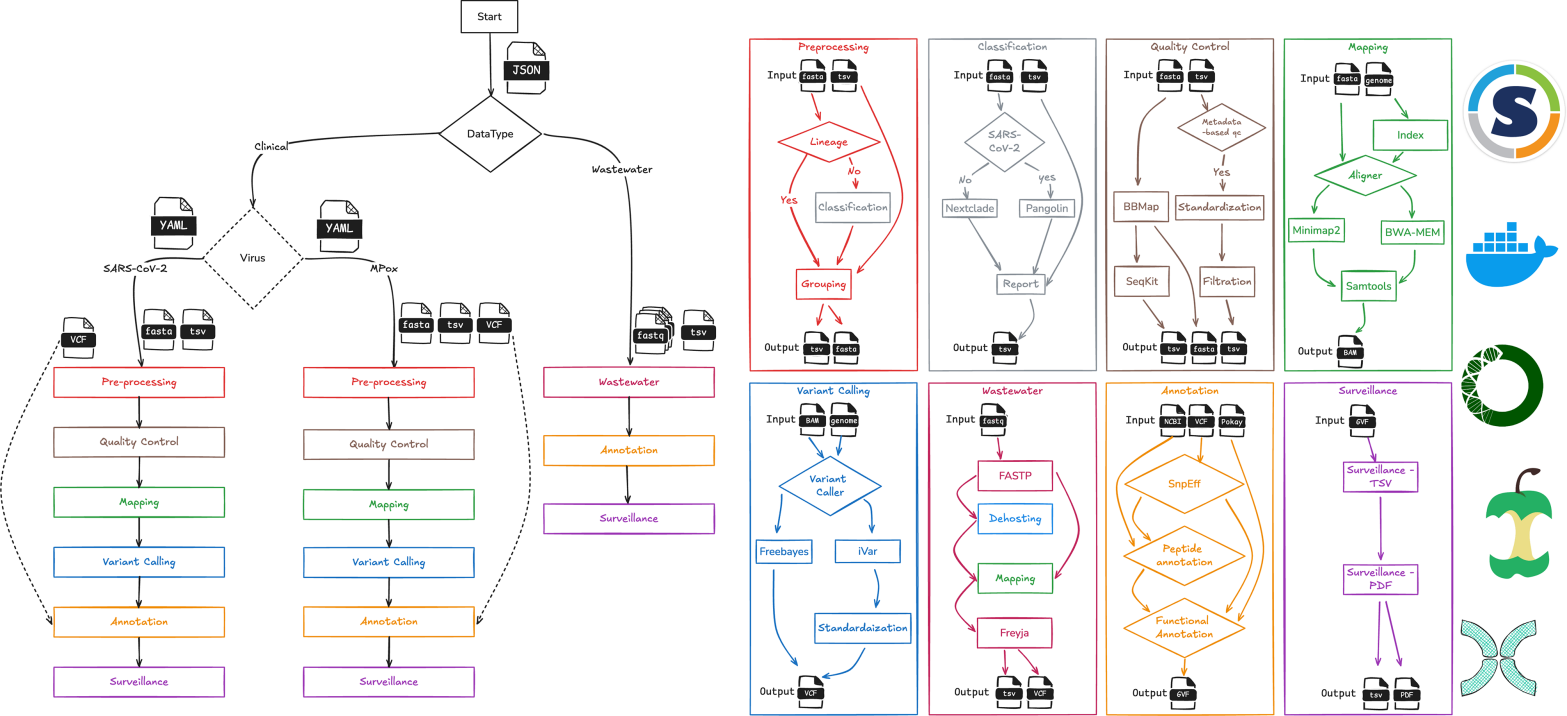
Design of genomics workflow. The right panel of the image shows a library of sub-workflows. Containerized environments and bioinformatics frameworks ensure modularity and reproducibility. The left panel displays how these sub-workflows are connected and controlled to process data from different viruses and data types, such as wastewater. The YAML and JSON files encode the parameters for sub-workflows.

The genomics workflow is implemented in the Nextflow workflow management system [15], following the nf-core guidelines [16]. The workflow is developed in a modular structure where each process or step of the analysis is implemented as a discrete module. Each module is containerized using software container technologies, including Singularity [26, 27], Docker [28], and BioConda [29]. The modules are connected in a particular order to form sub-workflows. The workflow contains >50 modules and 10 sub-workflows that perform a diverse set of analyses. The order and execution of these modules and sub-workflows are controlled by a main script tailored to each virus. The core parameters to run the workflow can be supplied in a YAML configuration file or provided directly on the command line. These parameters control the types of analyses to run and the arguments for a given analysis. At a minimum, the user needs to provide a FASTA file containing viral sequences or a VCF file as input. An accompanying metadata file containing information about the samples can be provided to assist in grouping samples based on user-defined criteria. A detailed structure of the workflow is presented in Fig. 3.

Before variant calling, the user can group samples by lineage/clades (default) or other metadata variables such as time, geographical location, or any other categorical variable in the supplied metadata file. If the lineage/clade information is not provided in the metadata file, the user can classify the samples using Pangolin [30] and Nextclade [31]. Pangolin is specifically designed for SARS-CoV-2, whereas Nextclade can be used for different viruses. Both tools are integrated into the workflow, and an in-house Python script is used to merge the classification report with the metadata file for downstream analysis.

To filter out low-quality or missing data, the workflow allows the user to perform quality filtering based on the grouped samples with (i) metadata-based filtering and (ii) sequence-based filtering. Several Python scripts are embedded in the workflow to perform metadata-based filtering. We also provide recommended default cut-off filters derived from our experience developing COVID-MVP. The script allows options to filter samples that (i) originate from nonhuman hosts; (ii) have a length shorter than the user-specified length (default: 95% of the length of reference sequence); (iii) lack a complete sample collection date (i.e. YYYY-MM-DD); and (iv) were collected outside the user-defined start and end dates (default: no limits). For the sequence-based quality filtering, the workflow uses two open-source tools, BBMAP [32] and SEQkit [33], that can filter out low-quality sequences, especially based on missing information (number of *N* bases).

We perform the variant calling to characterize the virus’s population structure. This process includes mapping sequences against the default reference genome (or provided by the user). The mapping process is carried out by one of the two open-source tools available in the workflow, minimap2 [34] and BWA-MEM [35]. The resulting BAM file is then used to call variants. Two variant-calling tools are currently available in the workflow: FreeBayes [36] and iVar [3]. FreeBayes produces a Genomic Variant Call Format (GVCF) file, which is further processed using an in-house Python script to filter out low-quality mutations and output a VCF file. For iVar, which produces a TSV file, we converted it to a VCF file using an in-house Python script. We have integrated a dedicated sub-workflow for processing the paired-end short reads to perform a similar analysis on the environmental samples (wastewater). The sub-workflow uses FastP [37] to trim/filter out low-quality reads. Like WGS sequences, quality-controlled reads are mapped to the reference genome using BWA-MEM or minimap2, resulting in a BAM file. Unlike WGS sequences, the wastewater samples may contain multiple co-circulating lineages/clades for a given virus. The workflow uses iVar to trim primers and call variants relative to the reference alignment file. This workflow embeds the Freyja workflow [38] to recover relative lineage abundances from mixed viral samples. Freyja is an open-source tool that uses lineage-determining mutational barcodes derived from the UShER global phylogenetic tree as a basis set to solve the de-mixing problem. It produces a TSV file, which is then converted to a VCF file using an in-house Python script.

The workflow uses several annotation sources, including the Pokay functional database, to profile and summarize mutations present in the supplied viral sequence data. The annotation sub-workflow has several modules and processes, some of which are used universally, while others are specific to the virus under study. One of the universally used tools is SnpEff [39], which is used to summarize mutations. SnpEff summarizes each mutation, including position, gene name, gene ID, and feature type. Variants and their annotations are recorded in GVF files. Multiple in-house Python scripts are embedded in the workflow to parse and annotate NCBI and RefSeq database records [40]. The GVF file is also annotated using the Pokay functional database and/or any other annotations formatted using the template provided (see the “Functional annotation framework” in the “Results” section). The resulting annotated GVF file is then used for visualization.

**Figure 4.**
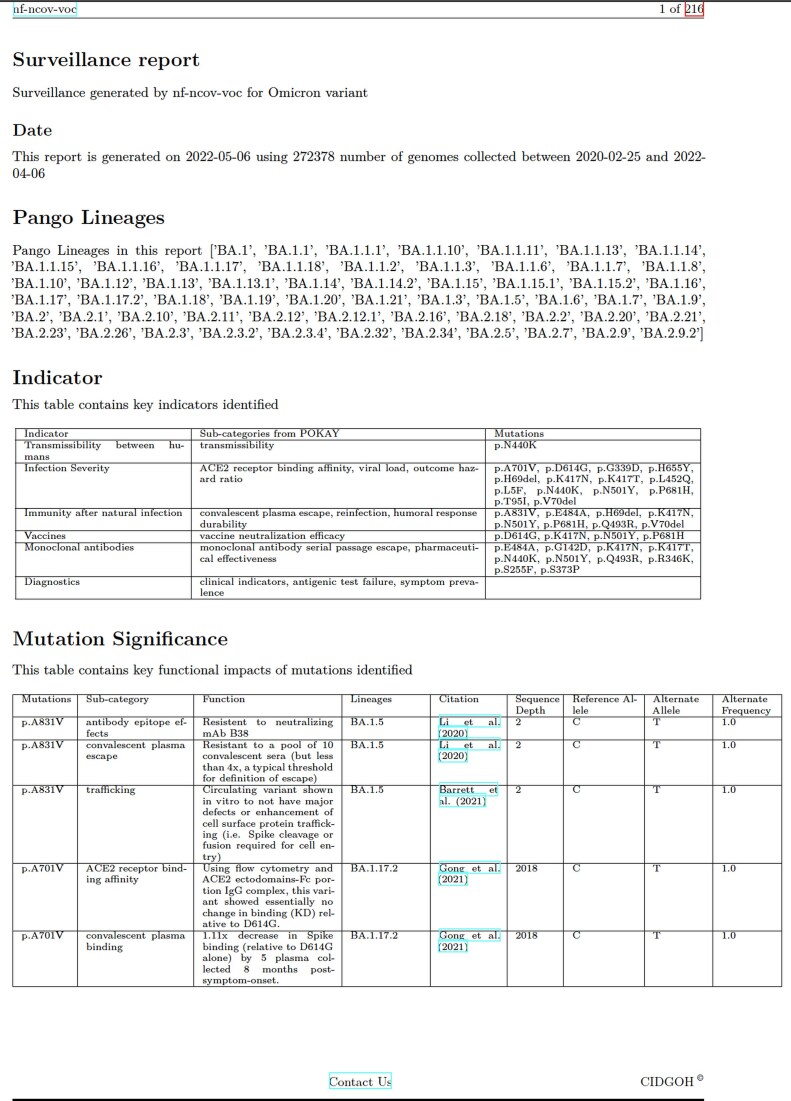
An example SARS-CoV-2 surveillance report with information on the number of genome sequences, identified viral (Pango) lineages, a table highlighting mutations associated with high-level surveillance indicators, and a table summarizing each mutation’s significance.

In addition to the GVF files produced by the workflow for visualization, the workflow also generates summarized surveillance reports. These surveillance reports are designed in two different formats: (i) A TSV file that contains each identified mutation and its corresponding functions and additional contextual information; and (ii) a PDF file containing human-readable summary tables. The TSV file can be used as input for integrating VIRUS-MVP with other surveillance and downstream analysis tools. Each column’s description, type, and source in the TSV file are available in our GitHub repository (see the “Availability and deployment” section). The surveillance reports are intended for end users who wish to incorporate them directly into their genomic surveillance reporting processes. The PDF file includes three sections (Fig. 4): the first section, Information on the variant or cluster of sequences section, includes (i) the range of dates for genomes based on the metadata field “*sample collection dates*” that are either provided by the user or extracted from the dataset; (ii) the number of genomes used in the analysis, to give context for the frequencies provided in section 3; and (iii) a list of Pangolin lineages or Nextclade-assigned clades identified in the dataset. The second section (*Indicator section*) contains a three-column table of disease indicators for public health surveillance, e.g. *transmissibility between humans, infection severity, etc*. (indicators can be specified for each run in an input file: see the “Availability and deployment” section), the table also includes functional categories (from Pokay) grouped by disease indicators (e.g. *ACE2 receptor binding affinity, viral load*, and *outcome hazard ratio* are classified under *infection severity)* and the mutations identified for each category and indicator. Finally, the third section (*mutation significance section)* consists of a table that provides each mutation’s prevalence, functional impact, hyperlinked citation for the study, lineage, number of sequences in the lineage from the dataset, reference allele, alternate allele, and alternate frequency. An example of a surveillance report in PDF format is available in the nf-ncov-voc repository (see the “Availability and deployment” section).

### Use cases

VIRUS-MVP’s flexible workflows and customizable datasets allow it to answer different questions related to viral evolution and genomic epidemiology. Here, we demonstrate two applications of VIRUS-MVP by leveraging data from the SARS-CoV-2 dedicated instance, which uses the Canadian national SARS-CoV-2 genomic surveillance dataset.

#### Longitudinal analysis

**Figure 5.**
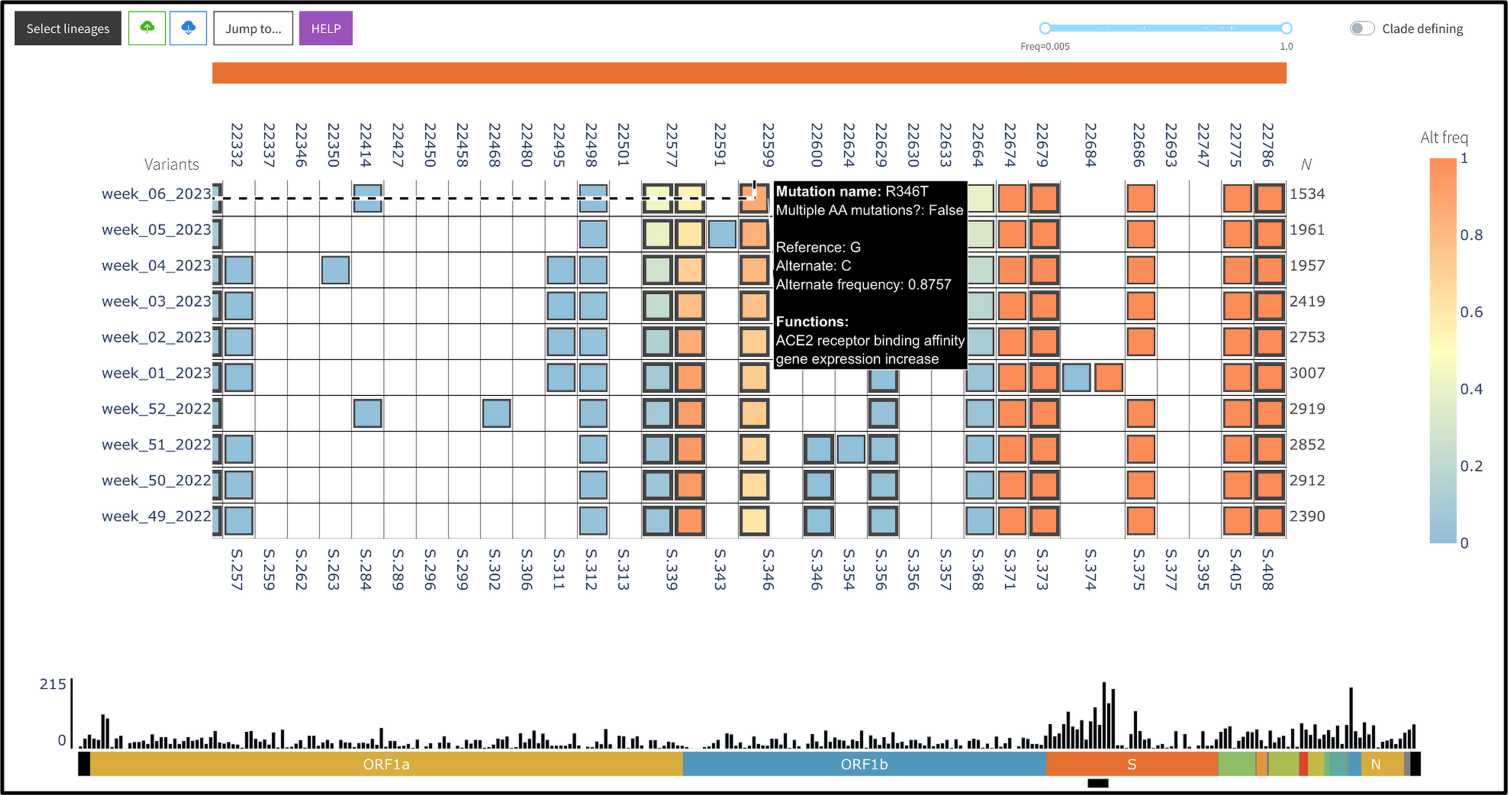
Longitudinal analysis of SARS-CoV-2 data from Canada (between Week 49 of 2022 and Week 06 of 2023) using VIRUS-MVP.

The custom grouping functionality of genomics workflow enabled us to perform a longitudinal analysis by organizing the dataset by epi-week rather than by lineage (Fig. 5). This approach allowed us to perform a week-by-week assessment of mutation frequency across the sample population. With adaptable axis settings in the visualization component, we could dynamically relabel the *Y*-axis with epi-week on the left and the number of isolates per week on the right. This flexibility facilitated the monitoring of significant mutations over time, independent of lineage assignments.

Applying this setup, we identified a notable increase in the S:R346T mutation in the spike protein, which appeared in Canada near the end of 2022. The S:R346T mutation enhances the virus’s binding affinity for ACE2 receptors on host cells, which can increase infection potential and impact virulence [41]. This mutation is prevalent in the Omicron sub-lineage XBB.1.5, initially detected in Canada around October 2022. The swift rise in XBB.1.5 cases corresponded with the increased frequency of S:R346T, underscoring VIRUS-MVP’s ability to identify mutations of interest early in their emergence.

By monitoring the growth trajectory of this mutation and its eventual establishment within an epidemiologically significant lineage, we gained insights into its potential selective advantage and fitness implications and demonstrated how VIRUS-MVP could support proactive genomic surveillance. This capability is particularly valuable for public health stakeholders, as it allows for early detection of mutations that may be associated with increased transmission or disease severity, potentially shaping research and intervention priorities.

#### Integrated wastewater and WGS analysis

**Figure 6.**
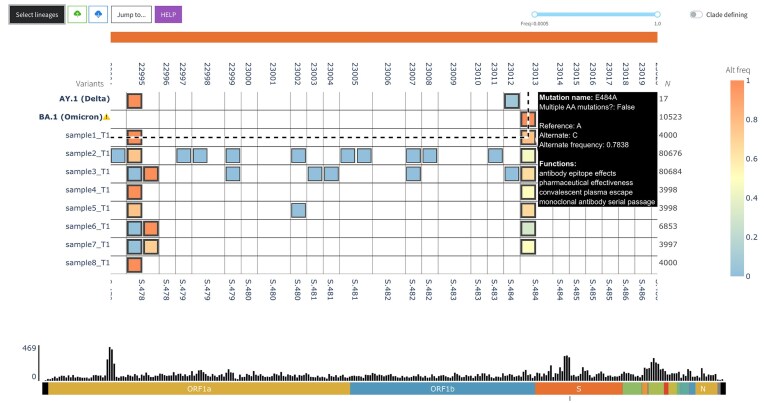
VIRUS-MVP was adapted to visualize SARS-CoV-2 wastewater sequencing data and compared to the Canadian genome sequencing data from the VirusSeq Data Portal in the same view.

Sequencing of viral genomes has been pivotal in COVID-19 surveillance, supporting pandemic response and enabling the study of viral evolution and prevalence. As part of a sustainable, long-term approach, a One-Health surveillance model is being implemented to integrate environmental surveillance with clinical monitoring systems [42, 43]. Environmental surveillance, particularly wastewater sampling, allows the detection of viral variants and mutations of interest within the community, providing early signals of viral dissemination across broader populations. Using VIRUS-MVP, wastewater samples—sequenced independently with a dedicated workflow—can be analyzed for mutations and variant prevalence, then directly compared with clinical genome sequences within the same visualization. This comparison helps determine if detected mutations in wastewater samples also appear in clinical specimens, enhancing mutation tracking. To validate this capability, we analyzed simulated wastewater amplicon sequencing data (Sutcliffe et al., 2023) alongside real genome sequencing data from the Canadian VirusSeq Data Portal in the same analysis view (Fig. 6). The use of simulated wastewater data allows us to know the ground truth.

For example, in a simulated sample (sample1_T1) composed of a 75:25 lineage ratio of BA.1 (Omicron) to AY.1 (Delta), VIRUS-MVP identified the S:E484A mutation with a 78% prevalence. Notably, S:E484A is specific to BA.1 (Omicron) and absent in AY.1 (Delta), supporting the simulated lineage ratio. This approach demonstrates VIRUS-MVP’s capability for early detection of key mutations in wastewater samples, which can be validated in clinical samples to monitor ongoing viral evolution.

## Discussion and future work

The rapid expansion of molecular surveillance during the COVID-19 pandemic underscored the power of genomic data to inform public health responses. Numerous tools emerged during this period, such as CoV-Spectrum [11], CovMT [10], and Outbreak.info [44], which offered real-time visualization, lineage tracking, and integration with epidemiological or clinical metadata. These platforms have been instrumental in enabling global genomic epidemiology and public communication of variant trends.

VIRUS-MVP contributes to this evolving ecosystem not as a replacement but as a complementary, toolkit-oriented solution. While existing platforms often support predefined datasets and centralized pipelines, VIRUS-MVP emphasizes flexibility: it enables users to process, analyze, and visualize their own viral genomic data through portable, reproducible workflows. This empowers research groups, surveillance teams, and public health labs to conduct localized or cloud-based analysis with control over their data and customizable reporting. By design, VIRUS-MVP embraces a modular, pathogen-agnostic architecture, allowing new viruses, features, and annotation types to be incorporated through configuration rather than redevelopment.

This focus on user adaptability sets VIRUS-MVP apart within the landscape. For instance, while CoV-Spectrum excels at aggregating global SARS-CoV-2 trends and Outbreak.info links mutation data to literature and clinical outcomes, VIRUS-MVP provides a complementary perspective: it offers a structured environment for local dataset processing, mutation-level annotation, and real-time visualization. Its integration of a curated annotation framework and interactive genome heatmaps makes it particularly valuable for labs seeking internal decision-making tools or researchers analyzing emerging pathogens in specific settings. See Table 1 below for a more specific comparison of key features across different tools.

**Table 1.** Comparison of key features of mutation-centric analysis across different tools and platforms

Feature	CovRadar	Cov-MT	CoV-Spectrum	Outbreak.info	VarEPS	VIRUS-MVP
Mutation focus	Spike gene mutations	All mutations	All mutations	All mutations	All mutations	All mutations
Data source	Global sample collections	Global sample collections	Global sample collections	Global sample collections	Global sample collections	Customizable dataset (user-defined sources)
Visualization	Frequencies over time and space	Evolution of the mutational landscape	Variant trends by region and time	Lineage and prevalence summaries	Static plots of mutation stats	Interactive heatmaps, gene navigation, functional overlays
Functional annotations	Not available	Severity-based summaries	Hospitalization/ mortality correlations	Literature-linked summaries for lineages	Functional effects based on prediction	Curated and NLP-mined annotations, custom annotation support
Accessibility	Web platform	Web platform	Web platform	Web platform	Web platform	Web-based and local native and Docker deployment
Open Source	Yes (GPL-3.0 License)	Yes (MIT License)	Yes (GPL-3.0 License)	Yes (GPL-3.0 License)	Not fully open	Yes (MIT License)
Customization	Limited	Limited	Limited	Limited	Limited	Highly configurable (genome, metadata, annotations, workflows)
Integration	Standalone tool	Standalone tool	Standalone tool	–	–	Standalone or integrated use; JSON exports for downstream tools

VIRUS-MVP supports portable deployment across computing environments, with containerization options such as Docker available for ease of installation and reproducibility, though we recognize the potential performance trade-offs associated with container-based setups. While containerized workflows may introduce modest overhead, especially for large-scale datasets, this design choice prioritizes reproducibility and ease of use across institutional systems where users may lack administrative privileges. To support users with more demanding performance requirements, we have included clear instructions for native installation and workflow customization via the VIRUS-MVP GitHub repository.

A core strength of VIRUS-MVP lies in its functional annotation capabilities, which integrate curated biological insights about specific mutations and their effects. This system connects genomic variants to ontology-based descriptors, protein-level impacts, and external references. However, the curation of these annotations presents ongoing challenges: the need for accuracy, scalability, and consistency across diverse viruses and data sources makes manual approaches time-intensive. To address this, we are actively exploring natural language processing (NLP) tools to semi-automate literature mining and summarization of mutation effects. In parallel, we are building a schema-driven, ontology-based annotation model to standardize how functional impacts are reported, facilitating reuse, integration, and pathogen generalization.

Another key area of development is wastewater surveillance, which presents unique challenges such as degraded RNA, fragmented genomes, and co-circulating viral lineages within a single sample. We have integrated a Freyja-based module within VIRUS-MVP to deconvolve these complex mixtures, leveraging lineage-defining mutation barcodes to estimate the relative abundance of viral lineages. These estimates are stored alongside mutation profiles and will soon be visualized in the dashboard to enhance interpretability in multi-lineage contexts. To manage sequencing noise and variable depth, users can also apply interactive mutation frequency thresholds, enabling tailored exploration of rare variants versus dominant strains. While we acknowledge that wastewater data remains more difficult to interpret than clinical whole-genome sequencing (WGS) data, VIRUS-MVP provides realistic, flexible tools to support environmental surveillance, and we are committed to adding more adaptability. Ongoing efforts include refining our visualization layer, enhancing metadata integration, and benchmarking mutation calling strategies tailored to low-abundance, mixed-population samples.

Looking ahead, we are expanding our support for other priority pathogens such as RSV, influenza, and HIV. Although this manuscript focuses on SARS-CoV-2 and mpox—our most complete and validated implementations—the architecture of VIRUS-MVP is designed to scale. Clear documentation, modular configuration, and open-source code ensure that users can extend the toolkit independently. We have developed extensive documentation for users who wish to use the tool for their other viruses or their own data; they can refer to the “Adapting the Workflow to a New Virus” section on the GitHub README. This community-driven, extensible approach reflects our broader philosophy: to empower users to build their own solutions using a shared foundation.

In summary, VIRUS-MVP provides a flexible, pathogen-agnostic, modular, and extensible genomics toolkit that complements existing global dashboards by supporting local and cloud-based analysis of viral genomes. Its integration of customizable workflows, interactive visualizations, and a curated functional annotation system enables a wide range of applications in research and public health. By continuing to invest in new annotation strategies, wastewater support, and pathogen expansions, we aim to keep VIRUS-MVP at the forefront of genomic surveillance for both current and emerging threats.

## Availability and deployment

We have deployed VIRUS-MVP on a cloud service using the docker-compose setup found on the application GitHub repository. The VIRUS-MVP docker image is built on top of the latest Python base image, and the resulting container is served through gunicorn [45]. To establish a secure SSL connection, we implemented an Nginx reverse proxy setup [46] external to the VIRUS-MVP GitHub repository.

For users seeking to deploy an application instance with upload functionality, the genomics workflow built within the deployed container also utilizes a Docker containerization strategy by default. This strategy was implemented due to the relatively slower performance of a Conda containerization strategy and difficulties with installing Singularity inside an existing Docker container. To avoid a Docker-in-Docker scenario leading to container escape, the docker-compose.yml file bind mounts the host docker socket. This Docker-based architecture makes VIRUS-MVP highly portable across various cloud environments. With containerized components, users can deploy VIRUS-MVP on any cloud provider that supports Docker, enabling flexible scaling and straightforward replication in new environments.

- VIRUS-MVP instance dedicated to SARS-CoV-2 data - https://virusmvp.org/covid-mvp
- VIRUS-MVP visualization source code, manual, and deployment - https://github.com/cidgoh/VIRUS-MVP and Zenodo (https://doi.org/10.5281/zenodo.16988800)
- VIRUS-MVP Genomics workflow source code - https://github.com/cidgoh/nf-ncov-voc and Zenodo (https://doi.org/10.5281/zenodo.16988885)
- Functional Database Pokay - https://github.com/nodrogluap/pokay
- Mutation Functional Annotation Contextual Data Specifications package https://github.com/cidgoh/pathogen-mutation-functionalannotation-package
- SARS-CoV-2 surveillance indicators for public health - https://github.com/cidgoh/nf-ncov-voc/tree/master/assets/ncov_surveillanceIndicators
- Surveillance report (TSV format description) - https://github.com/cidgoh/nf-ncov-voc/blob/master/docs/surveillance_report_description.md
- Surveillance report (PDF format example) - https://github.com/cidgoh/nf-ncov-voc/blob/master/docs/Omicron_surveillance_report.pdf
- All sequence data used in developing, testing, and generating the figure is available at https://virusseq-dataportal.ca
